# Holistic View and Novel Perspective on Ruminal and Extra-Gastrointestinal Methanogens in Cattle

**DOI:** 10.3390/microorganisms11112746

**Published:** 2023-11-10

**Authors:** Godson Aryee, Sarah M. Luecke, Carl R. Dahlen, Kendall C. Swanson, Samat Amat

**Affiliations:** 1 Department of Microbiological Sciences, North Dakota State University, Fargo, ND 58108, USA; godson.aryee@ndsu.edu (G.A.); sarah.luecke@ndsu.edu (S.M.L.); 2 Department of Animal Sciences, and Center for Nutrition and Pregnancy, North Dakota State University, Fargo, ND 58102, USA; carl.dahlen@ndsu.edu (C.R.D.); kendall.swanson@ndsu.edu (K.C.S.)

**Keywords:** methanogens, enteric methane emission, cattle, microbiome, holistic, extra-intestinal microbial communities, rumen

## Abstract

Despite the extensive research conducted on ruminal methanogens and anti-methanogenic intervention strategies over the last 50 years, most of the currently researched enteric methane (CH_4_) abatement approaches have shown limited efficacy. This is largely because of the complex nature of animal production and the ruminal environment, host genetic variability of CH_4_ production, and an incomplete understanding of the role of the ruminal microbiome in enteric CH_4_ emissions. Recent sequencing-based studies suggest the presence of methanogenic archaea in extra-gastrointestinal tract tissues, including respiratory and reproductive tracts of cattle. While these sequencing data require further verification via culture-dependent methods, the consistent identification of methanogens with relatively greater frequency in the airway and urogenital tract of cattle, as well as increasing appreciation of the microbiome–gut–organ axis together highlight the potential interactions between ruminal and extra-gastrointestinal methanogenic communities. Thus, a traditional singular focus on ruminal methanogens may not be sufficient, and a holistic approach which takes into consideration of the transfer of methanogens between ruminal, extra-gastrointestinal, and environmental microbial communities is of necessity to develop more efficient and long-term ruminal CH_4_ mitigation strategies. In the present review, we provide a holistic survey of the methanogenic archaea present in different anatomical sites of cattle and discuss potential seeding sources of the ruminal methanogens.

## 1. Introduction

Global warming is projected to have major consequences on food security worldwide, exacerbating the expected increase in food demand by 70% to 100% by 2050 due to the population growth [1,2,3]. Anthropogenic greenhouse gas (GHG) emissions, particularly CH_4_, are major contributors to global warming. Methane has a global warming potential approximately 28 times greater than CO_2_ [2]. The agricultural sector is considered to be one of the major sources of CH_4_ emissions, with ruminant animals, particularly domestic ruminants being significant contributors [4,5]. Ruminal methanogens are responsible for over 87% of total CH_4_ emissions from ruminants and about 26% in comparison to other sources of CH_4_ production in the environment [3,6]. Methane is produced in the rumen during the normal fermentation process by methanogenic archaea which use either CO_2_ and hydrogen (H_2_), methylamines or methanol, or acetate and H_2_ to produce CH_4_ [7]. Other microorganisms residing within the rumen such as bacteria, protozoa, and fungi can provide methanogens with excessive H_2_, either directly or indirectly, and thereby promote methanogenic activity [8,9]. Although utilizing excess H_2_ benefits ruminal fermentation by preventing H_2_ build-up and feedback inhibition, ruminal CH_4_ production represents 2–12% of gross energy loss [10]. Therefore, there is a need and impetus for developing approaches to mitigate CH_4_ emissions from ruminant livestock to reduce energy loss from animals while improving environmental health.

Over the last five decades, ruminal methanogens and anti-methanogenic intervention strategies have been extensively explored as a means of mitigating CH_4_ emissions [11]. These strategies involve alterations in animal management, dietary composition, and ruminal fermentation, as well as direct inhibition of the growth and metabolic activity of methanogens using anti-methanogenic compounds and substances [12,13]. However, most of these approaches have shown limited efficacy due to the complex nature of animal management and ruminal physiology [14,15]. Additionally, recent research has focused on reducing CH_4_ emissions from ruminants through genetic selection and manipulation of the ruminal microbiota, and the latter has become an active area of research due to advances in next-generation sequencing technologies [16]. While genomic selection could provide a long-term solution to CH_4_ emissions [17,18,19], the impact of the selected anti-methanogenic traits on ruminal nutrient metabolism, fermentation, and the microbial community is yet to be defined. The recent identification of a distinctive ruminal microbiota between cattle with high- and low-CH_4_-emitting phenotypes [20] suggests that the manipulation of the ruminal microbiota to mitigate livestock CH_4_ production may provide long-lasting solutions.

While most of the current research has focused on characterizing the taxonomic diversity and abundance of methanogenic archaea in the rumen and the potential role of the ruminal microbiome in methanogenesis, emerging evidence suggests that microbial communities associated with extra-ruminal sites of animals including the reproductive and respiratory tracts and mammary glands may harbor methanogenic archaeal species [7,21,22,23,24] and these extra-gastrointestinal methanogens may interact with ruminal methanogens. Some of the methanogenic taxa (e.g., *Methanobrevibacter* spp.) have been reported to be shared as core taxa across ruminal, respiratory, and reproductive tract-associated microbial communities in cattle [21,25]. This, coupled with increased appreciation of the microbiome–gut–organ (respiratory/reproductive) axis [25,26], highlights the possible existence of interactions between methanogenic archaea in the rumen with methanogens and microbiomes present in extra-gastrointestinal organs. Such interactions between the rumen and other organs may be responsible for seeding the rumen or other organs with methanogenic species and/or influencing metabolic activities of the ruminal methanogens. The potential seeding of the rumen with methanogens in the reproductive tract is further supported by the recent identification of methanogenic archaeal species in fetal fluid and intestines of bovine fetuses [21,27]. Thus, these new developments point out that focusing solely on the methanogens present in the rumen and developing CH_4_ mitigation strategies targeted at only ruminal methanogens could be too narrow of an approach. The ruminal and extra-gastrointestinal methanogens and their interactive effects on ruminal CH_4_ production should be considered. In this review, we first provide a holistic survey of the methanogenic archaea present in different anatomical sites of cattle. We then discuss potential seeding sources of the ruminal methanogenic archaea in cattle. In addition, we highlight some challenges and future research directions associated with studying ruminal and extra-gastrointestinal methanogens in cattle.

## 2. Brief Overview of Methanogens

Methanogens are a diverse group of microorganisms that produce CH_4_ as a metabolic byproduct from their energy conservation processes [28,29]. They are commonly present in a variety of environments including the digestive tracts of animals, predominantly ruminants, wetlands, and other anaerobic environments [11,30]. Methanogens are classified in the domain archaea and are phylogenetically diverse [31]. Methane production is an essential part of the global carbon cycle, accounting for approximately 18% of anthropogenic GHG emissions [32].

Methanogens are classified within the archaeal phyla *Euryarchaeota*, *Crenarchaeota*, and *Korarchaeota*. They are further subdivided into several orders and families based on their phylogenetic relationships and metabolic pathways [33,34]. Based on the substrates used to produce CH_4_, methanogens can be classified as hydrogenotrophic, acetoclastic, and methylotrophic methanogens [35,36]. The hydrogenotrophic class uses H_2_ for the reduction of CO_2_ into CH_4_. Examples of such methanogens are *Methanobrevibacter*, *Methanobacterium*, and *Methanomicrobiales.* The rumen is mainly inhabited by hydrogenotrophic methanogens [37]. The second class, acetoclastic methanogens which are most commonly present in freshwater sediments and anaerobic digestors, but use alcohols such as ethanol or 2-propanol as electron donors to produce CH_4_ [38,39,40,41,42]. The methylotrophic class which is predominant in freshwater and wetland soils [43,44,45] relies on methyl groups such as methanol and methylamines to produce CH_4_, and encompasses the order *Methanococcales* and *Methanosarcinales*. Of note, hydrogenotrophic methanogens are the focus of the present review as they are the main class of methanogens involved in enteric CH_4_ emissions from cattle. The other two classes of methanogens will be briefly discussed as potential environmental sources that could seed ruminal methanogens in cattle.

## 3. Main Methanogenic Species Present in the Rumen

The methanogenic community in the rumen of ruminant animals is dominated by two main phyla: Euryarchaeota and Crenarchaeota [46]. The Euryarchaeota phylum is the most abundant and taxonomically diverse group of methanogens in the rumen. This phylum includes the orders *Methanobacteriales*, *Methanomicrobiales*, *Methanosarcinales*, and *Methanocellales*. The Crenarchaeota phylum contains the order *Thermoproteales* and is present in some ruminants but is less abundant as compared to the Euryarchaeota phylum [47] (Table 1). *Methanobrevibacter* is the most abundant genus of ruminal methanogens in cattle and it belongs to Euryarchaeota phylum [47]. Multiple species of *Methanobrevibacter* including *M. ruminantium*, *M. smithii*, and *M. gottschalkii* have been reported in the rumen of cattle and other ruminant species (Table 1). The *Methanobrevibacter* spp. produce CH_4_ from H_2_ and CO_2_, and they can also utilize formate and acetate as alternative substrates [48]. Another common archaeal genus found in the rumen is *Methanosphaera* [49], and species within this genus consume H_2_ and CO_2_, as well as methanol and methylamines to produce CH_4_ [49]. Multiple species within *Methanosphaera* (e.g., *M. stadtmanae* and *M. cuniculi*) have also been detected in the rumen, and believed to contribute to the ruminal CH_4_ production [50,51]. Guzman and colleagues identified *Methanomicrobiales mobile*, *Methanoccocales votae*, and *Methanobrevibacter* spp. from the gastrointestinal tract of neonatal dairy calves sampled within five minutes of birth [52]. *Methanomassiliicoccus*, within the phylum Euryarchaeota, is a relatively recent discovered genus from the rumen and feces of ruminants [50]. This genus is unique in a way that it produces CH_4_ through the reduction of methanol and methylamines, rather than H_2_ and CO_2_ [50]. *Methanomassiliicoccus* has been suggested as a potential target for inhibiting ruminal CH_4_ emissions due to its ability to outcompete hydrogenotrophic methanogens [53]. Also, *Methanospirillum*, classified within the order *Methanomicrobiales* and the phylum Euryarchaeota, has been found in the rumen and feces of cattle and sheep [54]. Additionally, *Methanospirillum hungatei* and *Methanospirillum lacunae* are also present in the rumen [55]. Overall, the rumen is home to taxonomically and metabolically diverse methanogenic archaeal species.

## 4. Pro-and Anti-Methanogenic Ruminal Microorganisms

Methanogens undergo methanogenesis; an energy-intensive process that requires specific substrates and environmental conditions [68]. Syntrophic bacteria are among the most extensively studied microorganisms that enhance the activity of methanogens in the rumen. These bacteria form symbiotic associations with methanogens, by degrading complex organic matter to simpler compounds that methanogens can utilize [50,69]. For example, *Syntrophomonas wolfei*, *Syntrophobacter fumaroxidans*, and *Pelotomaculum thermopropionicum* can oxidize short chain fatty acids (SCFAs) to produce H_2_ and CO_2_, both of which are then used by methanogens to produce CH_4_ [70]. In addition to syntrophic bacteria, some other bacterial species may enhance the activity of methanogens through various mechanisms in the rumen. For example, exopolysaccharide-producing bacterial species can promote the aggregation of methanogens, creating microenvironments favoring methanogenesis [48]. Other bacteria species that produce secondary metabolites including formic acid and ethanol can also facilitate methanogenesis [71]. In addition, *Pelobacter* spp. and *Bacteroides* spp. can promote methanogenesis by enhancing the growth of specific methanogenic species [72].

While some bacteria promote methanogens, there are others in the rumen that can inhibit methanogenesis. For example, sulfate-reducing bacteria (SRB) such as *Desulfovibrio* spp. consume organic matter and produce hydrogen sulfide (H_2_S). Both SRB and methanogens compete for H_2_, as SRB requires it to reduce sulfate to sulfide. Because of this competition, the presence of SRB indirectly decreases CH_4_ production in the rumen [20]. Ruminal SRB utilize various forms of sulfur (S), including sulfate, sulfite, thiosulfate, and elemental S, as optional H_2_ sinks [73]. As the end-product of the sulfate reduction pathway, H_2_S can inhibit methanogenic activity, consequently reducing ruminal CH_4_ production [74]. In addition, ruminal *Prevotella*, *Fibrobacter*, and *Ruminococcus* have been reported to have negative correlation with methanogenic activity in dairy cows [14]. These bacteria are involved in the degradation of fiber and the production of propionate thereby competing with methanogens for H_2_. Fumarate-reducing and lactatic acid-producing bacteria (LAB) may also inhibit methanogens, which was suggested by a study where nitrate supplementation reduced CH_4_ emission in grazing steers by promoting fumarate-reducing bacteria and LAB [75]. This is further supported by Jenayathan and colleagues [12], who reported that direct-fed microbes comprising *Propionibacterium* and *Lactobacillus* spp. were able to mitigate CH_4_ emissions in sheep. The genus *Lactobacillus* has a long history of being used as a probiotic and has recently been proposed to mitigate ruminal CH_4_ emissions [76]. Other genera such as *Prevotella* and *Succinivibrio* have been negatively associated with ruminal methanogenesis [49,58,77]. The family *Succinivibrionaceae* is a key gut microbial member in the Tammar wallaby, which produce only 20% of the CH_4_ emissions of cattle [78]. This is because *Succinivibrionaceae* produce succinate through the fumarate–succinate pathway, which is an intermediate product of propionate, which is easily absorbed by the animal for energy [79].

Certain viral species can infect methanogens, called methanogenic viruses or methanophages. Methanophages against *Methanosarcina*, *Methanococcus*, and *Methanobacterium* [80,81,82] have been reported in methanogen abundant anoxic environments. Viruses that infect bacterial species can compete with methanogens for substrates [80] and have also been reported in methanogenic environments. Accordingly, it is plausible that viruses infecting methanogenic archaea or anti-methanogenic bacteria are present in the bovine rumen.

Methane produced in the rumen can be utilized by other ruminal microbial community members. Methanotrophic archaea, also known as methanotrophs, are a group of archaea that can utilize CH_4_ as the sole source of carbon and energy [83]. Under aerobic conditions, methanotrophs combine O_2_ and CH_4_ to form formaldehyde, which is then incorporated into organic compounds via the ribulose monophosphate pathway by type I methanotrophs (γ-proteobacteria) or the serine pathway by type II methanotrophs (α proteobacteria) [83]. The methanotrophic bacteria, including *Methylobacterium*, *Methylomonas* and *Methylomicrobium* genera, have been detected in the bovine rumen and have been reported to influence methanogens [84].

Fungi species have been shown to promote methanogenic activity by producing various enzymes such as cellulases and hemicellulases, and these enzymes can break down complex polysaccharides (cellulose and hemicellulose) into simpler compounds that can be utilized by methanogens [85]. Anaerobic fungi, such as *Neocallimastix* spp. and *Piromyces* spp., have been extensively studied for their ability to enhance the activity of methanogens in the rumen [85]. These fungi species are commonly found in the rumen and play a crucial role in the degradation of plant biomass [86]. As discussed above, it is apparent that the activities of methanogens in the rumen are interdependent on other microbes and their respective activities in the ruminal environment. Thus, understanding the interactions between the methanogens and other microorganisms within the rumen is important as such interactions could be harnessed for mitigating enteric methane emissions from cattle.

## 5. Methanogens in the Reproductive Tract: Vagina, Uterus, and Semen

**Figure 1:**
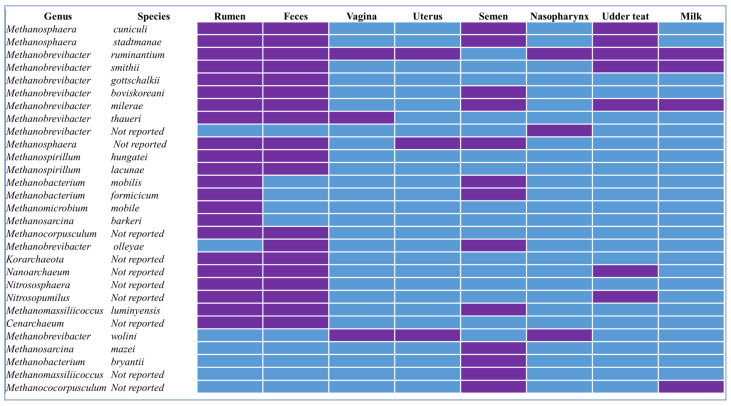
Presence (purple color) or absence (blue) of methanogenic archaeal species reported in different anatomical sites of cattle. This figure was generated based on the data presented in the references listed in Table 1 and Table 2.

Methanogen presence has been reported in both the lower and upper reproductive tracts of cattle (Figure 1, Table 2). The microbial community associated with the bovine vaginal tract has been relatively well characterized as compared to the cervical and uterine microbiota [87]. Overall, there is less species richness and community diversity in the vaginal microbiota as compared to the bovine gut, and this community has been shown to influence reproductive health and fertility of female cattle [87,88]. Although the archaeal members of the vaginal microbiome have not yet been as extensively characterized as the bacterial members, the presence of some methanogens in the cattle vagina have been reported from 16S rRNA gene amplicon sequencing studies. The *Methanobrevibacter* genus has been reported to be the predominant methanogen genus in the vaginal samples of Nellore cows [89]. We recently reported six amplicon sequence variants (ASVs) that were classified as *Methanobrevibacter* spp. (five of these taxa were unclassified at the species level, and the remaining one was *M. ruminantium*) present from vaginal swabs of both virgin yearling heifers and pregnant beef heifers [21]. The *Methanobrevibacter ruminantium* taxa was most frequently identified from the vaginal swab samples with greater abundance than the other *Methanobrevibacter* taxa [21].

While there are no culture-based studies reporting the isolation and identification of methanogens in the vaginal tract of cattle, methanogenic archaeal species have been cultured and isolated from the vaginal swabs [93] and urine specimens [94] of women with urinary tract infections. Belay and colleagues [93] were able to identify two different *Methanobrevibacter smithii* strains from the vaginal swabs of women diagnosed with bacterial vaginosis using traditional anaerobic culturing technique. Briefly, vaginal swabs were enriched in a medium containing various salts and additives, and yeast extract and tryptone in anaerobic serum tubes pressurized with H_2_-CO_2_ and supplemented with cysteine hydrochloride and Na_2_S·9H_2_O. Methane production in the serum tube was monitored via gas chromatography. Following enrichment, the cultures that produced CH_4_ gas were plated on medium with 1% Gelrite to isolate methanogens. The two methanogen isolates were taxonomically identified as *Methanobrevibacter smithii* PS and ALI based on the morphological, cultural, and immunological features. Likewise, Grine and colleagues [94] isolated *Methanobrevibacter smithii* strains from the urine samples of women suffering from urinary tract infections using a Hungate culture tube containing SAB medium, a versatile medium that supports the growth of most of methanogen species [95]. Based on their methanogenic archaeal culturing results from 383 urine specimens (61% of them from women) prospectively collected for diagnosing urinary tract infection, these authors suggest that *M. smithii* is part of the urinary microbiota of some individuals. Thus, both sequencing and culturing-based results obtained from bovine and human studies discussed above highlight that the microbial community in the bovine lower reproductive tract could harbor methanogenic archaea. The physiological function of methanogens in the lower reproductive tract of cattle remains to be explored even though some studies have suggested potential association of methanogens with reproductive health [21,89,96,97]. In the human vaginal tract, methanogens are known to play a significant role in the prevention of acid accumulation (increase in vaginal pH) which disrupts the vaginal microbiota in bacterial vaginosis patients [93,94,98].

The in-utero environment has long been viewed as sterile as the cervix was thought to prevent ascending bacteria from the lower reproductive tract into the uterus [99]. However, culture-independent high-throughput sequencing technologies have enabled the identification of commensal microbiota presence in the bovine uterus both during pregnancy and after parturition [100]. The potential role of the uterine microbiome in reproductive health, conception, and embryo development is increasingly appreciated [87,101,102]. Within the bovine uterine microbial community, methanogenic archaeal species have been reported. A sequencing-based study identified several methanogens in both pregnant and non-pregnant cows, including *Methanosphaera stadtmanae*, *Methanobrevibacter ruminantium*, and *Methanobacterium congolense* [89] (Table 2; Figure 1). Our lab has also identified *Methanobrevibacter ruminantium* and *Methanobrevibacter wolini*, which accounted for 0.05% of the total microbiota present in the uterus of virgin beef heifers (21 months old) [25]. We also observed greater abundance of *Methanobrevibacter ruminantium* (ASV330) in the uterine microbiota of beef cows that became pregnant than those that remained open following artificial insemination, suggesting a positive association of *Methanobrevibacter ruminantium* with fertility [103]. In contrast, methanogens have been shown to be present in the uterus of cows with uterine infections, suggesting a potential role in pathogenesis [104]. The functional and taxonomic characteristics of methanogens in the bovine uterus, like those found in the vagina, are yet to be fully explored.

Recent sequencing-based studies revealed that there are diverse and dynamic microbial communities residing within the bull reproductive tract [105]. Methanogenic archaeal species are identified as commensal microbiota associated with bull semen and the abundance of methanogens in the semen may have an association with bull fertility. For example, Koziol and colleagues identified *Methanobrevibacter*, *Methanosphaera*, and *Methanomassiliicoccus* as the predominant methanogens present in the semen of breeding beef bulls [59] using 16S rRNA gene amplicon sequencing. Likewise, *Methanocorpusculum* spp. was identified in bovine bull semen, and the relative abundance of this methanogenic genus was inversely correlated with seminal commensal genera *Ruminoccoceae* and *Rikenellaceae* RC9 Gut group [90]. Our research group detected more than a dozen of methanogenic archaeal taxa (listed in Figure 1; Table 2) from semen samples collected at three different stages of yearling beef bull development [67]. These studies together demonstrated that the male reproductive tract is colonized by methanogens. Although the functional features of the seminal microbiota including methanogenic archaeal members are yet to be characterized, a negative correlation of methanogenic archaeal abundance with bull fertility has been reported [59,90]. The bulls with low fertility had a greater abundance of seminal *Methanocorpusculum* as compared to bulls with high fertility [90]. Semen samples with a greater abundance of methanogens had significantly lower sperm motility compared to semen samples with lower levels of methanogens, suggesting a potential negative correlations between methanogens and sperm motility and development [59]. In a commercial beef cow-calf operation in the U.S., where natural breeding is used as a primary means to breed female cattle, one bull is expected to breed more than 20 female cattle [106]. The methanogens present in a single bull reproductive tract would be possible to transfer to many female reproductive tracts, and then ultimately to offsprings. Meanwhile, the bull semen could serve as transferring medium for methanogens between female cattle. Therefore, seminal methanogens and their transfer among female and offspring cattle deserve further research attention.

## 6. Methanogens in the Respiratory Tract

Bovine respiratory microbial communities, particularly bacterial microbiota in the upper and lower respiratory tracts have been well studied using both culturing and sequencing-based methods due to their role in protecting or predisposing animal to bovine respiratory disease (BRD), which is one of the costliest diseases affecting commercial feedlot cattle [107,108,109,110]. While most of these sequencing methods used to characterize bovine respiratory microbiota are mainly limited to the 16S rRNA amplicon (V4) and (V3–V4) sequencing (which is more specific to bacterial populations), several studies have reported the presence of methanogenic archaea in the upper respiratory tract (Table 2; Figure 1). Amat and colleagues reported the presence of the *Methanobrevibacter* genus in the nasopharynx of feedlot steers [21]. Several taxa within the Methanobrevibacter, including *Methanobrevibacter ruminantium*, have also been detected in the nasopharynx of virgin and pregnant beef cattle [21]. Two *Methanobrevibacter* species (*wolinii* and *ruminantium*) have also been reported in the nasopharyngeal microbiota of finishing feedlot heifers [25]. The lower airway is also colonized by a microbial community, and bacterial microbiota associated with the trachea [92,108,111] and lung tissue have been relatively well documented in cattle. However, presence of archaeal species in the lower airway has not been reported in any of these studies. Since the 16S rRNA gene (V4 region) was used by most of these studies, and neither archaeal-targeted amplicon sequencing nor shotgun metagenomic sequencing approaches have been employed to characterize the lung tissue samples, it is challenging to make a conclusive statement on the presence or absence of methanogenic archaea in the bovine lower respiratory tract.

However, evidence derived from human studies suggests that the lower respiratory tract may harbor methanogenic archaea. *Methanobrevibacter* spp. (*oralis* and *smithii*) have been cultured and isolated from sputum, trachea-bronchial, and broncho-alveolar samples collected from humans [112].

Methanogenic archaeal cell presence in the upper respiratory tract of cattle raises an important question about the survival mechanisms of methanogens in such an O_2_ rich environment given that archaeal species involved in methanogenesis are believed to be strict anaerobes. It is not uncommon to identify anaerobic bacterial species some of which are associated with the ruminal commensal microbiota such as *Ruminococcus* and *Thermodesulfovibrio* (sulfate-reducing bacteria) in the upper respiratory tract of cattle [113]. Anaerobic bacteria are predominant components of the upper respiratory tract, and mixed anaerobic–aerobic agents are often responsible for respiratory infections in humans [114]. Thus, despite the fact that some of the methanogenic species in the nasopharyngeal and lung tissue samples of cattle can be transient and from eructation and inhalation of a ruminal gas cap, the aerosols generated from the dust and soil particles [92,115,116], the airway mucosal surface of the cattle can harbor methanogens as part of their commensal microbiota. These airway-specific methanogens could have evolved to adapt and survive in the microenvironments along the respiratory tract, especially those parts with reduced O_2_ levels. One of the potential methanogen-promoting factors in the airway can be associated with biofilm-forming bacterial cells, as biofilms can provide localized anaerobic conditions. Biofilms can form in different areas of the respiratory tract [117]. In addition, the presence of oxygen-utilizing bacteria in the respiratory tract may support the growth of methanogens by consuming O_2_ and thereby creating anaerobic pockets where methanogens can persist [118,119]. Future culture-dependent studies are needed to isolate methanogens from the respiratory tract and explore metabolic features of these methanogens, and their interactions with the methanogens present in the rumen.

## 7. Methanogens in the Udder

The mammary gland is another site that has been reported to harbor relatively diverse and rich microbial communities [120]. Distinctive and site-specific microbial communities are present in various niches of the udder including the teat apex, teat canal, milk, and colostrum [120]. Some of these communities encompass archaeal members (Table 2; Figure 1). Guo and colleagues characterized the archaeal community shared between the maternal rumen and milk in grazing yak calves. They identified two archaeal phyla and 11 different common archaeal genera [53]. The primary archaeal phyla found on the skin of the teat were *Euryarchaeota* (76.8%) and *Thaumarchaeota* (23.1%) with the dominant genera being *Methanobrevibacter* (61.7%) and *Candidatus nitrocosmicus* (12.5%). Similar findings have been reported for the skin of dairy cows, where *Methanobrevibacter* and *Methanosphaera* were identified as the primary genera [91]. Species within *Methanobrevibacter*, including *M. ruminantium*, *M. smithii*, *M. millerae*, and some *Methanocorpusculum* spp. have also been detected in the bovine milk [23,53,91]. No data are available regarding the presence of methanogenic archaea in bovine colostrum. However, *Methanobrevibacter oralis* and *M. smithii* species have been found in human colostrum [121], suggesting that methanogenic archaea may also be present in the cattle colostrum.

Similarly to the respiratory tract, the presence of methanogens on teat skin and udder raises an important question regarding the strict anaerobic nature of these methanogens. *Methanobrevibacter* and *Candidatus nitrocosmicus* are known to be strict anaerobes and are typically unable to survive with exposure to ambient air for more than 10 min [122]. Therefore, their survival on the teat skin would be unlikely unless there is a wound present that creates an anaerobic environment suitable for methanogens. Further, research is needed to investigate the mechanisms and conditions that facilitate the presence of methanogens on the skin and explore their potential role in the overall microbial ecology of the teat as related to milk production.

## 8. Potential Seeding Sources of the Ruminal Methanogens

**Figure 2:**
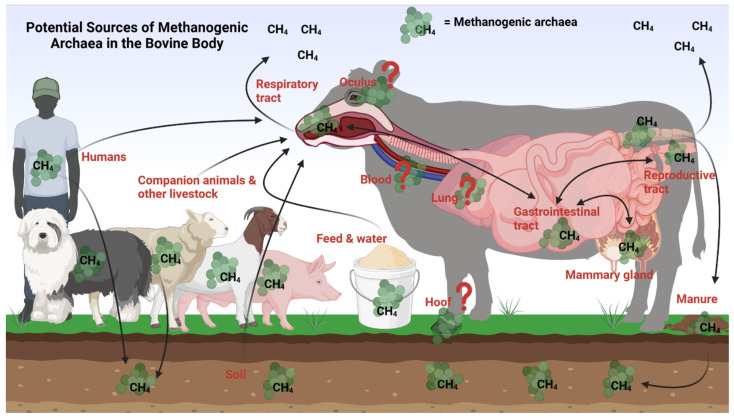
Holistic view of the bovine methanogens and potential sources of ruminal methanogens in cattle. Potential seeding sources of methanogenic archaea in the bovine body can include the soil, feed, water, farm laborers, other livestock species, or pets. Methanogens from the respiratory tract, reproductive tract, and mammary gland may seed the gastrointestinal tract, and vice versa. It is unclear if methanogens inhabit the oculus, blood, liver, lung, or hoof tissue, but the presence of other microorganisms at these sites could indicate the potential presence of methanogens here as well. Figure created using Biorender.com.

As illustrated in Figure 2, there are multiple potential seeding sources for the ruminal methanogenic archaea in cattle, and these sources could be the microbial communities present in the different anatomical sites of bovine body, and other external sources which will be discussed in detail below.

### 8.1. Within the Bovine Body

Ruminal methanogens have the potential to be seeded from the reproductive tract of the cow, particularly from the vagina, during calving. Our research team observed that certain methanogenic taxa are present in both the rumen and reproductive tract (vagina and uterus) of beef cattle [21,25]. This may suggest that there could be methanogenic archaea transfer between the gastrointestinal and reproductive tracts within the same animal. Likewise, the methanogenic archaeal species associated with the bovine upper respiratory tract may also be involved in introducing the rumen with methanogenic species. Our recent research revealed the presence of methanogenic taxa shared by the rumen and nasopharynx as core taxa [21,25]. Another important seeding source for the ruminal methanogens could be the udder and mammary gland of female cattle. As discussed above, methanogens can be present in bovine teat and milk. When neonatal calves are nursing, it becomes a direct route for transferring methanogens into the calf gut [93]. Additionally, bull semen harbors methanogens, and thus, it is highly likely that the sperm containing methanogens traveling through the female reproductive tract could potentially introduce methanogens into the uterus and reproductive system of the female [59]. Therefore, it would not be surprising to find the same genera of methanogens in both the sperm and milk and in the vagina, uterus, and gut of animals. In addition, diverse and dynamic bacterial microbiota has been reported to be present on the ocular surface of healthy newborn calves [105] and weaned beef calves [123], and cattle hooves are also home to commensal microbiota [105,124]. These studies have not reported the presence of methanogenic archaea in bovine eye and feet. However, until the absence of the methanogens from these sites is confirmed with studies using archaeal- or methanogen-specific amplicon sequencing or shotgun metagenomic sequencing, the possibility of the methanogenic archaeal exchange between ocular and hoof microbial communities with the bovine gut could not be ruled out.

### 8.2. Other Sources

There are several external sources that could introduce cattle rumen with methanogens (Figure 2). Among which, the pasture and soil may be the main external sources transferring methanogens to the cattle rumen [52,58]. Methanogens are commonly found in the soil [125,126]. The common and dominant genera of the soil-associated methanogens include *Methanoregula*, *Methanobacterium*, *Methanosarcina*, and *Methanolinea* spp. present in wetlands and water [127,128,129]. The presence of *Methanosarcina* and *Methanocella* spp. in cattle, sheep and swine grazing land [130], and wet soils and desert soils [131] have been documented. *Methanosarcina* spp. have been identified as the major methanogen in pasture soils compacted by cattle [22,132] and arable soils [133]. Cattle are known to consume approximately 350 kg of soil per cow per year through the so-called process of geophagia, which refers to the act of eating soil and dirt [134,135]. Thus, it is most likely that methanogens associated with soil can be transferred into the cattle rumen.

Methanogens could be transmitted to cattle via farm dust and dust particles. A study was conducted by Bønløkke and colleagues to investigate the exposure of livestock farmers to archaea [136]. For this, they analyzed the number of 16S rRNA gene copies from archaea and bacteria present in the personal filter samples obtained from 327 farmers working on 89 Danish farms including cattle and chicken farms. Both archaea and bacteria were detected in all types of farm environments. *Methanobrevibacter* and *Methanosarcina* species were found to be dominant in aerosols from both pig and cattle farms [136]. The aerosols likely consisted of a mixture of nasal fluid from farmers and other farm workers, as well as dust from the soil. When these aerosols are inhaled by cattle, they could potentially transfer methanogens into the airway systems and ultimately to the cattle rumen.

Farmers and farm workers could also be a source for introducing the cattle with methanogens. The human gut and other parts of the body harbor methanogens. *Methaninobrevibacter smithii* and *Methanobrevibacter oralis* are found in the intestine and sputum, while *Methanobrevibacter smithii* has been reported in bronchoalveolar [112], milk [137], and urine [94]. *Methanospaera stadmagnae* [138] and *Methanobrevibacter oralis* are associated with periodontal disease [112,139]. The methanogens present in the human body can be first transferred into the environment through spit, feces, and urine, and then ultimately to cattle.

Other animal species (e.g., sheep, chicken, pig, and dogs) raised on the same farm with cattle could inoculating cattle rumen with methanogens. Guindo et al. [60] conducted a study where fecal samples were analyzed from pigs, dogs, cats, sheep, and horses using methanogens targeted PCR. Seven different species of methanogens were present including *Methanobrevibacter smithii*, *Methanobrevibacter millerae* and *Methanomassiliicoccus luminyensis*, some of which are known to be present in the human digestive tract. *Methanobrevibacter smithii* were present in all the animal species studied [60]. Specifically, *Methanobrevibacter smithii* was present 50% of cases in pigs, 25% of cases in dogs, 16.7% of cases in cats, and 4.2% in both sheep and horses [60]. These findings indicate that *Methanobrevibacter smithii* is core archaeon shared by multiple animal species and humans, and it could be transferred between human and different animal species.

## 9. Challenges Associated with Studying the Ruminal Methanogens and Future Directions

In vitro culturing techniques allow researchers to isolate and study specific microorganisms in a controlled environment and can provide insights into the physiology and ecology of these organisms. The isolation and culturing of methanogens from the rumen is a particularly challenging task due to their strict anaerobic nature and growth requirements for special culturing media, culturing apparatus, and H_2_ gas supplementation, all of which have hindered the progress of isolation, and metabolic and genomic characterization of the methanogenic archaea associated with ruminal CH_4_ production [140,141]. While recent advances in culture-independent high-throughput sequencing techniques have enabled characterization of ruminal microbiota and the potential involvement in methanogenic activity, the information is mostly limited to the bacterial population of the ruminal microbial ecosystem as most of the sequencing is based on 16S rRNA gene amplicon sequencing, which is more specific to bacteria and captures only a small fraction of the archaeal population. Thus, to understand the complete taxonomic characterization of the methanogens and other archaeal populations in the rumen, and other extra-gastrointestinal sites of the bovine body, archaea-targeting amplicon sequencing such as 16S rRNA V2-V3 primers and *mcrA* gene-based primers should be applied [142]. To gain insights beyond the taxonomic properties of bovine methanogens, the metabolic features, and their interactions with other microorganisms such as fungi, bacteria and viruses, shotgun metagenomic sequencing should be performed. Also, comparative genomics on methanogenic isolates from different cattle body sites (e.g., respiratory, reproductive, ruminal and udder) should be performed to understand the genomic adaptative features of the methanogens from different anatomical sites and identify the seeding sources of the ruminal methanogens. Source tracking pipeline [143,144] can be applied to 16S rRNA gene or shotgun metagenomic sequences to identify main seeding sources of the methanogens in the rumen. Given the several external sources including the feed, soil and water consumed by cattle, and other farm animals and farmers who could exchange methanogens with cattle rumen and other body sites, a comprehensive survey of the methanogens present in these external sources and their interactions with ruminal methanogens in cattle warrants further research.

While these culture-independent sequencing methods can provide taxonomic composition and functional features of the methanogenic archaeal members and their interactions with other microbial partners in a particular niche, one of the limitations of these sequencing techniques is that they could not provide information on the viability of the methanogenic archaeal cells. Considering the anaerobic nature of methanogens and their presence in non-anaerobic body sites including respiratory and lower reproductive tracts of cattle, it is critical to use culture-dependent techniques to isolate methanogens from these sites and verify findings from metagenomic sequencing. It is plausible that some of the genomic DNA extracted from the samples associated with nasopharynx, lung tissue and vagina of cattle might be originated from transient and dead methanogens. Nevertheless, the identification of methanogenic archaeal genera and species with relatively high abundance, and consistently over the multiple sampling timepoints in bovine respiratory and reproductive tracts presented above, highlights that the methanogenic archaea could colonize and survive aerobic mucosal surfaces along the airway and urogenital tract of cattle. Thus, further research is warranted to isolate methanogens from extra-gastrointestinal tracts of cattle and explore the survival mechanisms of the methanogens in the environment where oxygen is present. Some anaerobic bacterial species can have evolved mechanisms that can either minimize the extent to which oxygen disrupts their metabolism [145,146] and/or rely on potential anaerobic and aerobic species’ co-existence mechanisms [147].

Emerging evidence from humans [146], bovine fetal fluids [21,27], and the fetal intestine [21,27,148], as well as the human fetal lung [149], suggests that microbial colonization of calves may begin in utero. This, coupled with rodent studies which demonstrate that fetal metabolic and nervous system development is impacted by the maternal microbiota during pregnancy [150,151], highlights the potential and extended role of the maternal microbiome in calf microbiome development. Recent studies reported the presence of methanogenic archaeal species in fetal fluids and fetal intestine at 4–8 months of gestation. Thus, these recent developments warrant a re-consideration of the timing and mechanisms involved in the first colonization of calf ruminal methanogens.

## 10. Conclusions

Methanogens are responsible for enteric CH_4_ emissions in cattle, which contribute to a significant amount of dietary energy loss to the host and GHG emissions. The species associated with ruminal CH_4_ production are not only present in the cattle rumen, but they could also present in extra-gastrointestinal organs of cattle such as the reproductive and respiratory tracts, udder, eye, and hoof. Recent sequencing-based studies revealed that certain methanogenic taxa are shared by the ruminal, respiratory, and reproductive tracts of cattle as core taxa, suggesting potential interactions between the ruminal and extra-intestinal methanogens. The seeding of the rumen with methanogenic archaea could be from many possible sources within the bovine body (e.g., reproductive, and respiratory tracts, and other microbial communities) and external sources such as pasture, soil, water, and farm animals. Therefore, a singular focus on the methanogens in the rumen may not be sufficient, and a holistic approach which takes into consideration the transfer of methanogens between ruminal, extra-gastrointestinal, and environmental microbial communities. Taxonomic, genomic, and metabolic characteristics of the methanogenic archaeal species in the rumen and other bovine body sites should be investigated to gain more holistic insights into the methanogens in cattle. It is anticipated that the holistic understanding of the methanogens in the rumen and their interactions with the extra-gastrointestinal methanogens, the identification of the seeding sources (both within and external), and the colonization timing of the ruminal methanogens are important for the development of more effective CH_4_ mitigation strategies in cattle.

## Figures and Tables

**Table 1:** Methanogenic archaeal species present in the rumen and feces of ruminant animals.

Phylum	Family	Genus	Species	Sequencing Method	Host	Reference
Rumen						
Euryarchaeota	*Methanobacteriaceae*	*Methanobrevibacter*	*ruminantium*, *smithii*, *gottschalkii*, *boviskoreani*, *milerae*	16S rRNA Sequencing V4	Korean native cattle	[56]
Euryarchaeota	*Methanobacteriaceae*	*Methanobrevibacter*	*ruminantium*, *smithii*, *gottschalkii*, *boviskoreani*, *milerae*	16S rRNA Sequencing V6–V8	Sheep	[57]
Euryarchaeota	*Methanobacteriaceae*	*Methanobrevibacter*	*ruminantium*, *smithii*, *gottschalkii*, *boviskoreani*, *milerae*, *thaueri*	16S rRNA Sequencing V4	Dairy cow	[58]
Euryarchaeota	*Methanobacteriaceae*	*Methanobrevibacter*	*Ruminantium*	16S rRNA Sequencing V4	Beef heifers	[21]
Euryarchaeota	*Methanobacteriaceae*	*Methanosphaera*	Not reported	16S rRNA	Ruminants	[50]
Euryarchaeota	*Methanobacteriaceae*	*Methanosphaera*	*stadtmanae*, *cuniculi*	16S rRNA Sequencing V4	Breeding bulls	[59]
Thermoplasmatota	*Methanomassiliicoccaceae*	*Methanomassiliicoccus*	*Luminyensis*	qPCR	Sheep, cow	[60]
Euryarchaeota	*Methanospirillaceae*	*Methanospirillum*	*hungatei* and *lacunae*	16S rRNA Sequencing V4	Dairy cow	[61]
Euryarchaeota	*Methanobacteriales*	*Methanobacterium*	*mobilis*, *formicicum*, *barkaeri*	16S &18S	Grazing cattle	[62]
Euryarchaeota	*Methanobacteriaceae*	*Methanomicrobium*	*Mobile*	16S rRNA Sequencing V1–V2, V2–V3, culturing	Sheep	[63]
Euryarchaeota	*Methanosarcinaceae*	*Methanosarcina*	*Barkeri*	16S rRNA Sequencing, culturing	Dairy cow	[64]
Euryarchaeota	*Methanocorpusculaceae*	*Methanocorpusculum*	Not reported	16S rRNA Sequencing V3–V4	Dairy cow	[65]
Nitrososphaerota	*Nitrososphaeraceae*	*Nitrososphaera*	Not reported	16S rRNA Sequencing V3–V4	Dairy cow	[65]
Thermoproteota	*Cenarchaeaceae*	*Cenarchaeum*	Not reported	Shotgun metagenomics	Dairy cattle	[66]
Nitrososphaerota	*Nitrosopumilaceae*	*Nitrosopumilus*	Not reported	Shotgun metagenomics	Dairy cattle	[66]
Korarchaeota	*Korarchaeales*	*Korarchaeota*	Not reported	Shotgun metagenomics	Dairy cattle	[66]
Nanoarchaeota	*Nanoarchaeaceae*	*Nanoarchaeum*	Not reported	Shotgun metagenomics	Dairy cattle	[66]
Feces						
Euryarchaeota	*Methanobacteriaceae*	*Methanosphaera*	*stadtmanae*, *cuniculi*	16S rRNA Sequencing V4	Breeding bulls	[59]
Euryarchaeota	*Methanospirillaceae*	*Methanospirillum*	*hungatei* and *lacunae*	16S rRNA Sequencing V4	Dairy cow	[60]
Euryarchaeota	*Methanocorpusculaceae*	*Methanocorpusculum*	Not reported	16S rRNA Sequencing V3–V4	Sika deer, Dairy cow	[61]
Euryarchaeota	*Methanobacteriaceae*	*Methanobrevibacter*	*smithii*, *millaerae*, *labreanum*, *aggregans*	PCR	Sheep	[57]
Euryarchaeota	*Methanobacteriaceae*	*Methanobrevibacter*	*smithii*, *millaerae*, *labreanum*, *aggregans*, *thaueri*	PCR	Dairy cow	[65]
Euryarchaeota	*Methanobacteriaceae*	*Methanobrevibacter*	*boviskoreoni*, *millerae*, *olleyae*, *ruminantium*, *wolini*	16S rRNA Sequencing V3–V4	Dairy cow	[66]
Euryarchaeota	*Methanobacteriaceae*	*Methanobrevibacter*	Not reported	16S rRNA Sequencing V3–V4	Dairy cow	[66]
Euryarchaeota	*Methanobacteriaceae*	*Methanosphaera*	*cuniculi*	16S rRNA Sequencing V3–V4	Dairy cow	[66]
Euryarchaeota	*Methanobacteriaceae*	*Methanosphaera*	Not reported	16S rRNA Sequencing V3–V4	Dairy cow	[66]
Euryarchaeota	*Methanobacteriaceae*	*Not reported*	Not reported	16S rRNA Sequencing V3–V4	Dairy cow	[66]
Euryarchaeota	*Methanosarcinaceae*	*Methanosarcina*	*Mazei*	16S rRNA Sequencing V3–V4	Dairy cow	[60]
Halobacterota	*Methanomicrobia*	*Methanococorpusculum*	Not reported	16S rRNA Sequencing V3–V4	Dairy cow	[60]
Korarchaeota	*Korarchaeales*	*Korarchaeota*	Not reported	Shotgun metagenomics	Dairy cow	[66]
Nanoarchaeota	*Nanoarchaeaceae*	*Nanoarchaeum*	Not reported	Shotgun metagenomics	Dairy cow	[66]
Nitrososphaerota	*Nitrososphaeraceae*	*Nitrososphaera*	Not reported	16S rRNA Sequencing V4	Dairy cow	[65]
Nitrososphaerota	*Nitrososphaeraceae*	*Nitrososphaera*	Not reported	16S rRNA Sequencing V3–V4	Dairy cow	[65]
Nitrososphaerota	*Nitrosopumilaceae*	*Nitrosopumilus*	Not reported	Shotgun metagenomics	Dairy cow	[65]
Thermoplasmatota	*Methanomassiliicoccaceae*	*Methanomassiliicoccus*	*luminyensis*	qPCR	Sheep, cow	[60]
Thermoplasmatota	*Methanomethylophilaceae*	*Methanomassiliicoccus*	Not reported	16S rRNA Sequencing V3–V4	Beef bull	[67]
Thermoproteota	*Cenarchaeaceae*	*Cenarchaeum*	Not reported	Shotgun metagenomics	Dairy cattle	[66]

**Table 2:** Methanogenic archaeal species present in the reproductive and respiratory tracts of cattle.

Phylum	Family	Genus	Species	Sequencing Platform	Host	Reference
Vagina						
Euryarchaeota	*Methanobacteriaceae*	*Methanobrevibacter*	*ruminantium*	16S rRNA Sequencing V3–V4	Beef heifers	[21]
Euryarchaeota	*Methanobacteriaceae*	*Methanobrevibacter*	*ruminantium*	16S rRNA Sequencing V3–V4	Beef heifers	[25]
Euryarchaeota	*Methanobacteriaceae*	*Methanobrevibacter*	*wolini*	16S rRNA Sequencing V3–V4	Beef heifers	[25]
Euryarchaeota	*Methanobacteriaceae*	*Methanobrevibacter*	not reported	16S rRNA Sequencing V3	Beef heifers	[89]
Uterus						
Euryarchaeota	*Methanobacteriaceae*	*Methanobrevibacter*	*ruminantium*	16S rRNA Sequencing V3–V4	Beef heifers	[25]
Euryarchaeota	*Methanobacteriaceae*	*Methanobrevibacter*	*wolini*	16S rRNA Sequencing V3–V4	Beef heifers	[25]
Euryarchaeota	*Methanobacteriaceae*	*Methanosphaera*	not reported	16S rRNA Sequencing V3–V4	Beef heifers	[25]
Semen						
Euryarchaeota	*Methanobacteriaceae*	*Methanosphaera*	*stadtmanae*, *cuniculi*	16S rRNA Sequencing V4	Beef bulls	[59]
Thermoplasmatota	*Methanomassiliicoccaceae*	*Methanomassiliicoccus*	*luminyensis*	16S rRNA Sequencing V4	Beef bulls	[59]
Crenarchaeota	*Nitrososphaeraceae*	Not reported	Not reported	16S rRNA Sequencing V3–V4	Beef bulls	[67]
Euryarchaeota	*Methanobacteriaceae*	*Methanobacterium*	*mobilis*, *formicicum*, *bryantii*,	16S rRNA Sequencing V4	Beef bulls	[59]
Euryarchaeota	*Methanobacteriaceae*	*Methanobrevibacter*	*boviskoreoni*, *millerae*, *olleyae*, *ruminatium*, *wolini*	16S rRNA Sequencing V3–V4	Beef bulls	[67]
Euryarchaeota	*Methanobacteriaceae*	*Methanobrevibacter*	Not reportd	16S rRNA Sequencing V3–V4	Beef bulls	[67]
Euryarchaeota	*Methanobacteriaceae*	*Methanosphaera*	*cuniculi*	16S rRNA Sequencing V3–V4	Beef bulls	[67]
Euryarchaeota	*Methanobacteriaceae*	*Methanosphaera*	Not reported	16S rRNA Sequencing V3–V4	Beef bulls	[67]
Euryarchaeota	*Methanobacteriaceae*	*Not reported*	Not reported	16S rRNA Sequencing V3–V4	Beef bulls	[67]
Euryarchaeota	*Methanosarcinaceae*	*Methanosarcina*	*mazei*	16S rRNA Sequencing V3–V4	Beef bulls	[67]
Thermoplasmatota	*Methanomethylophilaceae*	*Methanomassiliicoccus*	Not reported	16S rRNA Sequencing V3–V4	Beef bulls	[67]
Halobacterota	*Methanomicrobia*	*Methanococorpusculum*	Not reported	16S rRNA Sequencing V3–V4	Beef bulls	[67]
Halobacterota	*Methanomicrobia*	*Methanococorpusculum*	Not reported	16S rRNA Sequencing V3–V4	Beef bulls	[90]
Milk						
Euryarchaeota	*Methanobacteriaceae*	*Methanobrevibacter*	*ruminantium*, *smithii*, *milerae*	Shotgun metagenomics	Dairy cow	[60,91]
Euryarchaeota	*Methanocorpusculaceae*	*Methanocorpusculum*	Not reported	Shotgun metagenomics	Dairy cow	[91]
Nasopharynx						
Euryarchaeota	*Methanobacteriaceae*	*Methanobrevibacter*	*ruminantium*	16S rRNA Sequencing V3–V4	Beef heifers	[25]
Euryarchaeota	*Methanobacteriaceae*	*Methanobrevibacter*	*wolinii*	16S rRNA Sequencing V3–V5	Beef heifers	[25]
Euryarchaeota	*Methanobacteriaceae*	*Methanobrevibacter*	Not reported	16S rRNA Sequencing V4	Beef steers	[92]
Udder teat						
Euryarchaeota	*Methanobacteriaceae*	*Methanobrevibacter*	*ruminantium*, *smithii*, *milerae*	Shotgun metagenomics	Dairy cow	[60,91]
Euryarchaeota	*Methanobacteriaceae*	*Methanosphaera*	*stadtmanae*, *cuniculi*	Shotgun metagenomics	Dairy cow	[91]
Nitrososphaerota	*Nitrosopumilaceae*	*Nitrosopumilus*	Not reported	Shotgun metagenomics	Yak calves	[53]
Nanoarchaeota	*Nanoarchaeaceae*	*Nanoarchaeum*	Not reported	Shotgun metagenomics	Yak calves	[53]

## Data Availability

No new data were used for the research described in the article.
